# Supplementary material for: Autophagosomes fuse to phagosomes and facilitate the degradation of apoptotic cells in Caenorhabditis elegans
Source: eLife. 2022 Jan 4;11:e72466. doi: 10.7554/eLife.72466 (PMC8769646; doi:10.7554/eLife.72466)
Supplement: Figure 1—source data 1. [file elife-72466-fig1-data1.docx]

**Numerical data for Figure 1J – mCherry::lgg-1 and mCherry::lgg-2 signal over time**

|  | **Genotype** | |
| --- | --- | --- |
| **Time(min)** | ***Wild-Type mCherry::lgg-1*** | ***Wild-Type mCherry::lgg-2*** |
| 0 | 1.000 | 1 |
| 2 | 0.852 | 1.671 |
| 4 | 0.852 | 2.186 |
| 6 | 0.741 | 1.660 |
| 8 | 0.593 | 1.336 |
| 10 | 0.630 | 1.078 |
| 12 | 0.852 | 1.268 |
| 14 | 0.630 | 1.213 |
| 16 | 2.222 | 1.984 |
| 18 | 1.963 | 1.884 |
| 20 | 1.963 | 2.566 |
| 22 | 2.000 | 2.544 |
| 24 | 2.333 | 3.192 |
| 26 | 2.407 | 3.394 |
| 28 | 2.741 | 3.696 |
| 30 | 2.778 | 4.345 |
| 32 | 2.852 | 4.434 |
| 34 | 3.296 | 5.217 |
| 36 | 3.667 | 5.128 |
| 38 | 4.593 | 5.385 |
| 40 | 4.963 | 5.463 |
| 42 | 4.741 | 6.414 |
| 44 | 4.704 | 6.526 |
| 46 | 4.889 | 7.756 |
| 48 | 5.556 | 8.125 |
| 50 | 6.259 | 8.170 |
| 52 | 6.963 | 8.841 |
| 54 | 6.963 | 10.060 |
| 56 | 6.815 | 11.593 |
| 58 | 7.407 | 13.327 |
| 60 | 8.111 | 12.902 |
| 62 | 8.815 | 13.304 |
| 64 | 9.333 | 12.957 |
| 66 | 10.852 |  |
