# Supplementary material for: Autophagosomes fuse to phagosomes and facilitate the degradation of apoptotic cells in Caenorhabditis elegans
Source: eLife. 2022 Jan 4;11:e72466. doi: 10.7554/eLife.72466 (PMC8769646; doi:10.7554/eLife.72466)
Supplement: Figure 2—source data 1. [file elife-72466-fig2-data1.docx]

**Numerical data for figure 2G – mCherry::LGG-1 Signal intensity over time.**

|  | **Genotype** | | | |
| --- | --- | --- | --- | --- |
| **Time(min)** | **Wild-Type** | ***atg-7(bp411)*** | ***atg-13(bp414)*** | ***epg-8(bp251)*** |
| 0 | 1.000 | 1.000 | 1.000 | 1.000 |
| 2 | 0.852 | 1.022 | 1.160 | 1.019 |
| 4 | 0.852 | 1.179 | 0.840 | 0.981 |
| 6 | 0.741 | 1.067 | 0.840 | 1.076 |
| 8 | 0.593 | 1.112 | 0.840 | 1.095 |
| 10 | 0.630 | 1.045 | 0.947 | 0.943 |
| 12 | 0.852 | 1.112 | 0.867 | 0.924 |
| 14 | 0.630 | 1.089 | 0.760 | 1.019 |
| 16 | 2.222 | 0.978 | 0.653 | 1.019 |
| 18 | 1.963 | 1.000 | 0.840 | 1.057 |
| 20 | 1.963 | 0.911 | 0.867 | 1.076 |
| 22 | 2.000 | 1.224 | 0.813 | 1.057 |
| 24 | 2.333 | 1.089 | 0.947 | 1.076 |
| 26 | 2.407 | 0.933 | 1.027 | 0.924 |
| 28 | 2.741 | 1.022 | 1.187 | 1.153 |
| 30 | 2.778 | 1.313 | 1.267 | 0.962 |
| 32 | 2.852 | 1.291 | 1.293 | 1.057 |
| 34 | 3.296 | 1.425 | 1.347 | 0.866 |
| 36 | 3.667 | 1.358 | 1.507 | 1.134 |
| 38 | 4.593 | 1.246 | 1.613 | 1.019 |
| 40 | 4.963 | 1.313 | 1.720 | 0.962 |
| 42 | 4.741 | 1.403 | 2.013 | 0.866 |
| 44 | 4.704 | 1.157 | 1.720 | 0.943 |
| 46 | 4.889 | 1.201 | 2.493 | 1.210 |
| 48 | 5.556 | 1.380 | 2.147 | 0.943 |
| 50 | 6.259 | 1.268 | 2.093 | 1.134 |
| 52 | 6.963 | 1.246 | 1.960 | 1.153 |
| 54 | 6.963 | 1.313 | 1.987 | 1.076 |
| 56 | 6.815 | 1.358 | 2.200 | 1.019 |
| 58 | 7.407 | 1.224 | 2.387 | 1.172 |
| 60 | 8.111 |  | 2.547 | 0.752 |
| 62 | 8.815 |  | 2.493 | 1.076 |
| 64 | 9.333 |  | 2.867 | 1.000 |
| 66 | 10.852 |  | 3.160 |  |

**Numerical data for figure 2I - mCherry::LGG-2 signal intensity over time**

|  | **Genotype** | | | |
| --- | --- | --- | --- | --- |
| **Time(min)** | **Wild-Type** | ***atg-7(bp411)*** | ***atg-13(bp414)*** | ***epg-8(bp251)*** |
| 0 | 1 | 1 | 1 | 1 |
| 2 | 1.671 | 0.876 | 0.959 | 0.963 |
| 4 | 2.186 | 0.943 | 0.912 | 1.054 |
| 6 | 1.660 | 0.947 | 0.898 | 0.828 |
| 8 | 1.336 | 1.053 | 0.918 | 0.928 |
| 10 | 1.078 | 1.032 | 0.925 | 0.877 |
| 12 | 1.268 | 1.018 | 0.912 | 0.914 |
| 14 | 1.213 | 1.117 | 1.034 | 0.908 |
| 16 | 1.984 | 1.071 | 1.129 | 0.946 |
| 18 | 1.884 | 1.110 | 1.075 | 0.860 |
| 20 | 2.566 | 1.283 | 1.116 | 0.788 |
| 22 | 2.544 | 1.261 | 1.000 | 0.874 |
| 24 | 3.192 | 1.258 | 1.102 | 0.842 |
| 26 | 3.394 | 1.290 | 1.000 | 0.928 |
| 28 | 3.696 | 1.286 | 1.014 | 0.862 |
| 30 | 4.345 | 1.385 | 0.891 | 1.075 |
| 32 | 4.434 | 1.488 | 0.986 | 1.192 |
| 34 | 5.217 | 1.459 | 0.993 | 1.212 |
| 36 | 5.128 | 1.516 | 0.986 | 1.221 |
| 38 | 5.385 | 1.611 | 1.027 | 1.218 |
| 40 | 5.463 | 1.678 | 1.000 | 1.292 |
| 42 | 6.414 | 1.901 | 0.918 | 1.224 |
| 44 | 6.526 | 2.007 | 0.973 | 1.332 |
| 46 | 7.756 | 1.876 | 0.871 | 1.453 |
| 48 | 8.125 | 1.933 | 0.939 | 1.347 |
| 50 | 8.170 | 2.064 | 0.952 | 1.694 |
| 52 | 8.841 | 2.113 | 0.932 | 1.785 |
| 54 | 10.060 | 2.276 | 1.048 | 1.972 |
| 56 | 11.593 | 2.304 | 1.109 | 1.777 |
| 58 | 13.327 | 2.053 | 0.932 | 1.831 |
| 60 | 12.902 | 2.304 | 1.095 | 2.103 |
| 62 | 13.304 | 2.092 | 1.109 | 1.880 |
| 64 | 12.957 | 2.113 | 1.054 | 2.006 |
