# Supplementary material for: Autophagosomes fuse to phagosomes and facilitate the degradation of apoptotic cells in Caenorhabditis elegans
Source: eLife. 2022 Jan 4;11:e72466. doi: 10.7554/eLife.72466 (PMC8769646; doi:10.7554/eLife.72466)
Supplement: Figure 2—source data 2. [file elife-72466-fig2-data2.docx]

**Numerical data and statistical analysis for figure 2H – Relative mCherry::LGG-1 signal intensity at 60min-post engulfment.**

|  | **Genotype** | | | |
| --- | --- | --- | --- | --- |
| **Sample** | **Wild-Type** | ***atg-7(bp411)*** | ***atg-13(bp414)*** | ***epg-8(bp251)*** |
| 1 | 3.99 | 0.7 | 0.7 | 0.5 |
| 2 | 5.76 | 0.8 | 0.7 | 0.8 |
| 3 | 6.61 | 0.8 | 0.8 | 0.8 |
| 4 | 6.62 | 1 | 1 | 0.8 |
| 5 | 6.88 | 1 | 1.1 | 1 |
| 6 | 7.03 | 1 | 1.1 | 1 |
| 7 | 8.44 | 1 | 1.2 | 1 |
| 8 | 8.88 | 1 | 1.5 | 1 |
| 9 | 9.02 | 1.1 | 1.8 | 1.1 |
| 10 | 9.39 | 1.2 | 1.8 | 1.4 |
| 11 | 10 | 1.4 | 1.9 | 1.4 |
| 12 | 11.8 | 1.4 | 2.1 | 1.4 |
| 13 | 11.81 | 1.6 | 2.3 | 1.5 |
| 14 | 12.32 | 1.8 | 4.1 | 1.5 |
| 15 | 19.42 | 2.3 | 13.8 | 2 |
| **Mean** | **9.198** | **1.20666667** | **2.39333333** | **1.14666667** |

| **Comparison** | **P-Value** |
| --- | --- |
| WT vs *atg-7* | 8.7632E-07 |
| WT vs *atg-13* | 1.1056E-05 |
| WT vs *epg-8* | 7.8892E-07 |

**Numerical data and statistical analysis for figure 2J - Relative mCherry::LGG-2 signal intensity at 60min-post engulfment**

|  | **Genotype** | | | |
| --- | --- | --- | --- | --- |
| **Sample** | **Wild-Type** | ***atg-7(bp411)*** | ***atg-13(bp414)*** | ***epg-8(bp251)*** |
| 1 | 4.2 | 0.8 | 0.8 | 0.6 |
| 2 | 4.2 | 0.8 | 1 | 1 |
| 3 | 4.4 | 1 | 1 | 1.3 |
| 4 | 5 | 1.1 | 1 | 1.6 |
| 5 | 5.4 | 1.1 | 1.1 | 1.8 |
| 6 | 6.3 | 1.2 | 1.2 | 1.9 |
| 7 | 6.8 | 1.2 | 1.2 | 1.9 |
| 8 | 7 | 1.3 | 1.3 | 2.2 |
| 9 | 7.1 | 1.7 | 1.4 | 2.3 |
| 10 | 7.3 | 1.7 | 1.4 | 2.5 |
| 11 | 9 | 1.8 | 1.8 | 2.9 |
| 12 | 9.4 | 1.8 | 1.9 | 3.1 |
| 13 | 15.2 | 1.9 | 1.9 | 6.9 |
| 14 | 17 | 1.9 | 2 | 11.6 |
| 15 | 17.1 | 2 | 4.1 | 12 |
| **Mean** | **8.36** | **1.42** | **1.54** | **3.57333333** |

| **Comparison** | **P-Value** |
| --- | --- |
| WT vs *atg-7* | 3.4034E-05 |
| WT vs *atg-13* | 3.4736E-05 |
| WT vs *epg-8* | 0.00338503 |
