# Supplementary material for: Autophagosomes fuse to phagosomes and facilitate the degradation of apoptotic cells in Caenorhabditis elegans
Source: eLife. 2022 Jan 4;11:e72466. doi: 10.7554/eLife.72466 (PMC8769646; doi:10.7554/eLife.72466)
Supplement: Figure 5—source data 1. [file elife-72466-fig5-data1.docx]

**Numerical data for figure 5A & B – Summary of cell corpse count and statistical analysis of *atg* mutants at 2-fold stage**

| **Strain** | **Genotype** | **# cell corpses** | **P value compare to WT** |
| --- | --- | --- | --- |
| N2 | *Wild-Type* | 11.3±1.8(n=15) |  |
| GK738 | *lgg-1(tm3489)* | 17.3±3.7(n=15) | 1.82177E-05 |
| FX14610 | *lgg-2(tm5755)* | 16.6±2.9(n=14) | 8.92891E-06 |
| ZH2831 | *lgg-1(tm3489); lgg-2(tm5755)* | 21.1±2.5(n=15) | 3.29861E-12 |
| HZ1687 | *atg-9(bp564)* | 18.7±2.8(n=17) | 9.34313E-10 |
| VC893 | *atg-18(gk378)* | 21.6±1.3(n=10) | 3.04806E-14 |
| HZ1684 | *atg-3(bp412)* | 20.7±3.2(n=15) | 1.97914E-09 |
| HZ1686 | *atg-7(bp411)* | 18.2±2.5(n=15) | 3.85841E-09 |
| CB369 | *unc-51(e369)* | 19.8±2.3(n=16) | 4.13092E-12 |
| HZ1683 | *atg-2(bp576)* | 18.7±2.8(n=13) | 9.68418E-08 |
| HZ1688 | *atg-13(bp414)* | 17.8±2.0(n=15) | 5.36748E-08 |
| HZ1691 | *epg-8(bp251)* | 19.8±1.9(n=15) | 8.85946E-13 |
