# Supplementary material for: Autophagosomes fuse to phagosomes and facilitate the degradation of apoptotic cells in Caenorhabditis elegans
Source: eLife. 2022 Jan 4;11:e72466. doi: 10.7554/eLife.72466 (PMC8769646; doi:10.7554/eLife.72466)
Supplement: Figure 5—figure supplement 1—source data 1. [file elife-72466-fig5-figsupp1-data1.docx]

**Numerical data for Figure 5 Figure supplement 1 – Summary of cell corpse count and statistical analysis of *atg* mutants at 1.5-fold stage**

|  | **Genotype** | | |
| --- | --- | --- | --- |
| **Sample** | ***lgg-1(tm3489)*** | ***lgg-1(tm3489), gfp::lgg-1*** | ***lgg-1(tm3489), mCherry::lgg-1*** |
| 1 | 21 | 17 | 20 |
| 2 | 18 | 13 | 11 |
| 3 | 25 | 13 | 14 |
| 4 | 17 | 14 | 13 |
| 5 | 18 | 12 | 14 |
| 6 | 16 | 12 | 20 |
| 7 | 16 | 15 | 17 |
| 8 | 18 | 13 | 16 |
| 9 | 18 | 14 | 14 |
| 10 | 16 | 17 | 18 |
| 11 | 19 | 16 | 11 |
| 12 | 17 | 17 | 14 |
| 13 | 21 | 13 | 15 |
| 14 | 17 | 16 | 14 |
| 15 | 24 | 15 | 16 |
| **Mean** | **18.73333333** | **14.46666667** | **15.13333333** |
| **P-Value** |  | **4.9134E-05** | **0.00140589** |

|  | **Genotype** | | |
| --- | --- | --- | --- |
| **Sample** | ***lgg-2(tm5755)*** | ***lgg-2(tm5755), gfp::lgg-2*** | ***lgg-2(tm5755), mCherry::lgg-2*** |
| 1 | 16 | 15 | 12 |
| 2 | 15 | 13 | 14 |
| 3 | 20 | 10 | 13 |
| 4 | 16 | 14 | 14 |
| 5 | 14 | 13 | 14 |
| 6 | 17 | 13 | 13 |
| 7 | 19 | 16 | 15 |
| 8 | 20 | 14 | 15 |
| 9 | 19 | 12 | 14 |
| 10 | 20 | 13 | 14 |
| 11 | 16 | 14 | 14 |
| 12 | 18 | 14 |  |
| 13 | 16 |  |  |
| 14 | 18 |  |  |
| 15 | 18 |  |  |
| **Mean** | **17.46666667** | **13.41666667** | **13.81818182** |
| **P-Value** |  | **2.03194E-06** | **2.10792E-06** |
