# Supplementary material for: Autophagosomes fuse to phagosomes and facilitate the degradation of apoptotic cells in Caenorhabditis elegans
Source: eLife. 2022 Jan 4;11:e72466. doi: 10.7554/eLife.72466 (PMC8769646; doi:10.7554/eLife.72466)
Supplement: Figure 6—source data 1. [file elife-72466-fig6-data1.docx]

**Numerical data for Figure 6F – The time it takes to engulf C3 cell corpses in each of the following mutant phenotypes.**

|  | **Genotype** | | | | |
| --- | --- | --- | --- | --- | --- |
| **Sample** | **Wild-Type** | ***atg-7(bp411)*** | ***lgg-2(tm5755)*** | ***lgg-1(tm3489)*** | ***rab-7(ok511)*** |
| 1 | 4 | 4 | 4 | 4 | 3 |
| 2 | 4 | 4 | 6 | 4 | 6 |
| 3 | 4 | 4 | 6 | 4 | 6 |
| 4 | 4 | 4 | 6 | 4 | 6 |
| 5 | 4 | 4 | 6 | 4 | 6 |
| 6 | 6 | 6 | 6 | 6 | 6 |
| 7 | 6 | 6 | 6 | 6 | 9 |
| 8 | 6 | 6 | 6 | 6 |  |
| 9 | 6 | 6 | 6 | 6 |  |
| 10 | 6 | 6 | 6 | 6 |  |
| 11 | 6 | 6 | 8 | 6 |  |
| 12 | 6 | 6 | 8 | 6 |  |
| 13 | 8 | 6 | 8 | 8 |  |
| 14 |  | 8 | 10 |  |  |
| 15 |  | 8 | 12 |  |  |
| **Mean** | **5.384** | **5.6** | **6.933** | **5.384** | **6** |
| **Min** | **4** | **4** | **4** | **4** | **3** |
| **Max** | **8** | **8** | **12** | **8** | **9** |
