# Supplementary material for: Autophagosomes fuse to phagosomes and facilitate the degradation of apoptotic cells in Caenorhabditis elegans
Source: eLife. 2022 Jan 4;11:e72466. doi: 10.7554/eLife.72466 (PMC8769646; doi:10.7554/eLife.72466)
Supplement: Figure 6—source data 2. [file elife-72466-fig6-data2.docx]

**Numerical data for Figure 6G - The time it takes to degrade C3 cell corpses in each of the following mutant phenotypes.**

|  | Genotype | | | | |
| --- | --- | --- | --- | --- | --- |
| **Sample** | **Wild-Type** | ***atg-7(bp411)*** | ***lgg-2(tm5755)*** | ***lgg-1(tm3489)*** | ***rab-7(ok511)*** |
| 1 | 44 | 40 | 38 | 56 | 63 |
| 2 | 46 | 44 | 48 | 56 | 78 |
| 3 | 46 | 50 | 50 | 58 | 90 |
| 4 | 49 | 54 | 54 | 61 | 93 |
| 5 | 50 | 54 | 58 | 64 | 102 |
| 6 | 52 | 56 | 60 | 65 | 108 |
| 7 | 52 | 58 | 60 | 66 | 120 |
| 8 | 54 | 58 | 62 | 66 |  |
| 9 | 54 | 58 | 62 | 66 |  |
| 10 | 54 | 64 | 64 | 69 |  |
| 11 | 55 | 66 | 66 | 74 |  |
| 12 | 56 | 70 | 66 | 75 |  |
| 13 | 56 | 72 | 74 | 76 |  |
| 14 | 56 | 72 | 78 | 86 |  |
| 15 | 58 | 74 | 90 | 88 |  |
| 16 | 60 | 92 |  | 93 |  |
| 17 |  |  |  | 139 |  |
| **Mean** | **52.625** | **61.375** | **62** | **74** | **93.4** |
| **Min** | **44** | **40** | **38** | **56** | **63** |
| **Max** | **60** | **92** | **90** | **139** | **120** |
