# Supplementary material for: Autophagosomes fuse to phagosomes and facilitate the degradation of apoptotic cells in Caenorhabditis elegans
Source: eLife. 2022 Jan 4;11:e72466. doi: 10.7554/eLife.72466 (PMC8769646; doi:10.7554/eLife.72466)
Supplement: Figure 7—source data 1. [file elife-72466-fig7-data1.docx]

**Numerical data for Figure 7C – Percentage distribution of single and double-labeled puncta in the LGG-labeled population in the engulfing cells for C1, C2, and C3.**

|  | **% of Puncta population** | | |
| --- | --- | --- | --- |
| **Sample** | **LGG-1^+^ LGG-2^-^** | **LGG-1-**  **LGG-2+** | **LGG-1^+^ LGG-2^+^** |
| 1 | 41.176 | 38.235 | 20.588 |
| 2 | 46.667 | 40.000 | 13.333 |
| 3 | 41.176 | 29.412 | 29.412 |
| 4 | 31.034 | 44.828 | 24.138 |
| 5 | 33.333 | 50.000 | 16.667 |
| 6 | 33.333 | 38.095 | 28.571 |
| 7 | 36.364 | 31.818 | 31.818 |
| 8 | 23.529 | 52.941 | 23.529 |
| **Mean** | **35.827** | **40.666** | **23.507** |
| **SD** | **7.188** | **8.220** | **6.423** |
