# Supplementary material for: Autophagosomes fuse to phagosomes and facilitate the degradation of apoptotic cells in Caenorhabditis elegans
Source: eLife. 2022 Jan 4;11:e72466. doi: 10.7554/eLife.72466 (PMC8769646; doi:10.7554/eLife.72466)
Supplement: Figure 8—source data 1. [file elife-72466-fig8-data1.docx]

**Numerical data for Figure 8E – Percentage of distribution of LGG-1^+^ or LGG-2^+^ puncta that are also RAB-7+ in the cytoplasm of the engulfing cells C1, C2, and C3.**

|  | **% of Puncta population** | |
| --- | --- | --- |
| **Sample** | **LGG-1^+^ RAB-7^-^** | **LGG-1^-^ RAB-7^+^** |
| 1 | 58.33 | 54.55 |
| 2 | 60 | 57.14 |
| 3 | 63.64 | 57.14 |
| 4 | 63.64 | 58.33 |
| 5 | 70 | 63.64 |
| 6 | 70 | 66.67 |
| 7 | 70 | 66.67 |
| 8 | 70 | 70 |
| 9 | 70 | 76.92 |
| **Mean** | **66.179** | **63.451** |
| **SD** | **4.818** | **7.333** |

**Numerical data for Figure 8F – Percentage of distribution of LGG-1^+^ or LGG-2^+^ puncta that are also RAB-7+ on the surfaces of the phagosomes (C1, C2, and C3) 2-min before the autophagosome-phagosome fusion occurs.**

|  | **% of Puncta population** | |
| --- | --- | --- |
| **Sample** | **LGG-1^+^ RAB-7^-^** | **LGG-1^-^ RAB-7^+^** |
| 1 | 100 | 100 |
| 2 | 100 | 100 |
| 3 | 100 | 100 |
| 4 | 100 | 100 |
| 5 | 100 | 100 |
| 6 | 100 | 100 |
| 7 | 100 | 100 |
| 8 | 100 | 100 |
| 9 | 100 | 100 |
