# Supplementary material for: Autophagosomes fuse to phagosomes and facilitate the degradation of apoptotic cells in Caenorhabditis elegans
Source: eLife. 2022 Jan 4;11:e72466. doi: 10.7554/eLife.72466 (PMC8769646; doi:10.7554/eLife.72466)
Supplement: Figure 8—source data 2. [file elife-72466-fig8-data2.docx]

**Numerical data for Figure 8G – The time(min) it takes for mCherry::lgg-1 to fuse to the phagosome.**

|  | **Genotype** | | |
| --- | --- | --- | --- |
| **Sample** | ***Wild-Type*** | ***rab-7(ok511)*** | ***vps-18(tm1125)*** |
| 1 | 10 | >64 | 22 |
| 2 | 10 | >64 | 22 |
| 3 | 12 | >68 | 22 |
| 4 | 12 | >68 | 22 |
| 5 | 12 | >78 | 26 |
| 6 | 12 | >78 | 28 |
| 7 | 12 | >84 | 28 |
| 8 | 14 | >88 | 28 |
| 9 | 14 | >114 | 34 |
| 10 | 14 |  | 36 |
| 11 | 14 |  | 38 |
| 12 | 16 |  | 42 |
| 13 | 18 |  | 46 |
| 14 | 24 |  | 50 |
| 15 | 28 |  | 64 |
| 16 |  |  | 76 |

**Numerical data for Figure 8H – The time(min) it takes for mCherry::lgg-2 to fuse to the phagosome.**

|  | **Genotype** | | |
| --- | --- | --- | --- |
| **Sample** | ***Wild-Type*** | ***rab-7(ok511)*** | ***vps-18(tm1125)*** |
| 1 | 10 | >72 | 30 |
| 2 | 10 | >92 | 42 |
| 3 | 12 | >98 | 42 |
| 4 | 14 | >106 | 48 |
| 5 | 16 | >110 | 50 |
| 6 | 16 | >112 | 54 |
| 7 | 16 | >112 | 58 |
| 8 | 18 | >114 | 66 |
| 9 | 18 | >114 | >82 |
| 10 | 18 |  | >84 |
| 11 | 20 |  | >90 |
| 12 | 20 |  | >92 |
| 13 | 20 |  |  |
| 14 | 22 |  |  |
| 15 | 26 |  |  |
| 16 |  |  |  |
