# Supplementary material for: Autophagosomes fuse to phagosomes and facilitate the degradation of apoptotic cells in Caenorhabditis elegans
Source: eLife. 2022 Jan 4;11:e72466. doi: 10.7554/eLife.72466 (PMC8769646; doi:10.7554/eLife.72466)
Supplement: Figure 9—source data 1. [file elife-72466-fig9-data1.docx]

**Numerical data Figure 9F – mNG::LGG-1 signal intensity over time.**

|  | **Genotype** | | |
| --- | --- | --- | --- |
| **Time (min)** | **Wild-Type** | ***ced-1 (e1735)*** | ***dyn-1 (n4039)*** |
| 0 | 1.000 | 1.000 | 1.000 |
| 2 | 1.071 | 0.983 | 0.970 |
| 4 | 0.964 | 0.957 | 1.090 |
| 6 | 0.929 | 0.894 | 0.860 |
| 8 | 0.893 | 1.034 | 1.000 |
| 10 | 0.929 | 0.974 | 0.910 |
| 12 | 0.429 | 1.086 | 0.940 |
| 14 | 1.786 | 0.813 | 1.060 |
| 16 | 4.964 | 1.026 | 1.200 |
| 18 | 5.429 | 0.974 | 1.090 |
| 20 | 5.750 | 0.948 | 1.030 |
| 22 | 6.250 | 1.095 | 1.260 |
| 24 | 7.429 | 0.940 | 1.600 |
| 26 | 7.429 | 1.118 | 1.830 |
| 28 | 8.786 | 1.060 | 1.890 |
| 30 | 8.750 | 0.966 | 2.030 |
| 32 | 9.679 | 1.086 | 1.940 |
| 34 | 10.429 | 1.118 | 2.370 |
| 36 | 13.107 | 1.066 | 2.370 |
| 38 | 14.714 | 1.149 | 2.260 |
| 40 | 20.393 | 1.072 | 2.570 |
| 42 | 24.286 | 1.155 | 2.540 |
| 44 | 26.250 | 1.184 | 2.740 |
| 46 | 26.821 | 1.201 | 3.060 |
| 48 | 32.250 | 1.121 | 3.430 |
| 50 | 33.393 | 1.201 | 3.710 |

**Numerical data for Figure 9G – mNG::LGG-2 signal intensity over time.**

|  | **Genotype** | | |
| --- | --- | --- | --- |
| **Time (min)** | **Wild-Type** | ***ced-1 (e1735)*** | ***dyn-1 (n4039)*** |
| 0 | 1.000 | 1.000 | 1.000 |
| 2 | 0.991 | 1.000 | 0.970 |
| 4 | 0.995 | 0.949 | 0.850 |
| 6 | 1.117 | 0.923 | 0.800 |
| 8 | 1.239 | 0.923 | 0.550 |
| 10 | 1.153 | 0.872 | 0.710 |
| 12 | 1.140 | 0.974 | 0.720 |
| 14 | 1.185 | 0.974 | 0.580 |
| 16 | 1.207 | 0.821 | 0.620 |
| 18 | 1.509 | 0.949 | 0.570 |
| 20 | 1.824 | 0.769 | 0.630 |
| 22 | 1.793 | 0.949 | 0.820 |
| 24 | 2.081 | 1.000 | 0.860 |
| 26 | 2.324 | 0.872 | 0.910 |
| 28 | 2.410 | 0.949 | 0.920 |
| 30 | 2.405 | 0.949 | 1.000 |
| 32 | 2.568 | 0.692 | 1.050 |
| 34 | 3.360 | 1.000 | 0.970 |
| 36 | 3.653 | 0.769 | 1.060 |
| 38 | 4.054 | 1.000 | 1.170 |
| 40 | 4.225 | 0.897 | 1.310 |
| 42 | 5.126 | 0.846 | 1.180 |
| 44 | 5.018 | 0.949 | 1.230 |
| 46 | 5.446 | 0.821 | 1.490 |
| 48 | 5.622 | 0.974 | 1.450 |
| 50 | 5.622 | 0.923 | 1.740 |
