# Supplementary material for: Autophagosomes fuse to phagosomes and facilitate the degradation of apoptotic cells in Caenorhabditis elegans
Source: eLife. 2022 Jan 4;11:e72466. doi: 10.7554/eLife.72466 (PMC8769646; doi:10.7554/eLife.72466)
Supplement: Figure 9—source data 2. [file elife-72466-fig9-data2.docx]

**Numerical data for Figure 9I – Recruitment of mNG::LGG-1 signal intensity over time.**

|  | **Genotype** | | |
| --- | --- | --- | --- |
| **Time (min)** | **Wild-Type** | ***ced-1 (e1735)*** | ***dyn-1 (n4039)*** |
| 0 | 1.0000 | 1.0000 | 1.0000 |
| 2 | 1.2154 | 1.0053 | 1.0153 |
| 4 | 1.6548 | 1.0487 | 1.0353 |
| 6 | 1.8419 | 0.9247 | 0.9949 |
| 8 | 2.3251 | 0.9468 | 1.0020 |
| 10 | 3.0675 | 0.9842 | 0.9598 |
| 12 | 3.4314 | 1.0636 | 0.9937 |
| 20 |  | 1.0195 | 0.9311 |
| 30 |  | 1.1274 | 0.9536 |
| 40 |  | 1.1577 | 0.9668 |

**Numerical data for Figure 9J – Recruitment of mNG::LGG-2 signal intensity over time.**

|  | **Genotype** | | |
| --- | --- | --- | --- |
| **Time (min)** | **Wild-Type** | ***ced-1 (e1735)*** | ***dyn-1 (n4039)*** |
| 0 | 1 | 1 | 1 |
| 2 | 1.35 | 0.91 | 1.02 |
| 4 | 1.45 | 0.98 | 1.1 |
| 6 | 1.5 | 0.92 | 1.04 |
| 8 | 1.5 | 0.91 | 1.05 |
| 10 | 1.7 | 0.9 | 1.16 |
| 12 | 1.5 | 0.83 | 1.2 |
| 20 |  | 0.98 | 1.16 |
| 30 |  | 0.95 | 1.2 |
| 40 |  | 0.83 | 1.34 |
