# Supplementary material for: Autophagosomes fuse to phagosomes and facilitate the degradation of apoptotic cells in Caenorhabditis elegans
Source: eLife. 2022 Jan 4;11:e72466. doi: 10.7554/eLife.72466 (PMC8769646; doi:10.7554/eLife.72466)
Supplement: Figure 9—source data 3. [file elife-72466-fig9-data3.docx]

**Numerical data and statistical analysis for Figure 9K – Relative mNG::LGG-1 signal intensity at 50min-post engulfment.**

|  | Genotype | | |
| --- | --- | --- | --- |
| **Sample** | **Wild-Type** | ***ced-1 (e1735)*** | ***dyn-1 (n4039)*** |
| 1 | 2.9 | 1 | 0.56 |
| 2 | 4 | 1 | 0.56 |
| 3 | 4.3 | 1.45 | 0.7 |
| 4 | 4.8 | 1.62 | 0.82 |
| 5 | 4.9 | 1.7 | 1.03 |
| 6 | 4.9 | 1.9 | 1.08 |
| 7 | 5.1 | 1.97 | 2 |
| 8 | 6 | 2.06 | 2.14 |
| 9 | 6.3 | 2.08 | 2.74 |
| 10 | 6.53 | 2.32 | 3.03 |
| 11 | 6.6 | 2.33 | 3.26 |
| 12 | 6.7 | 2.55 | 4.27 |
| 13 | 7 | 2.63 | 5.57 |
| 14 | 7.2 | 4.24 | 5.94 |
| 15 | 8.2 | 5.95 | 6 |
| **Mean** | **5.695** | **2.32** | **2.646** |

| **Comparison** | **P-Value** |
| --- | --- |
| WT vs *ced-1* | 1.8684E-07 |
| WT vs *dyn-1* | 5.7862E-05 |

**Numerical data and statistical analysis for Figure 9L - Relative mNG::LGG-2 signal intensity at 50min-post engulfment.**

|  | **Genotype** | | |
| --- | --- | --- | --- |
| **Sample** | **Wild-Type** | ***ced-1 (e1735)*** | ***dyn-1 (n4039)*** |
| 1 | 3.21 | 0.86 | 0.75 |
| 2 | 4.16 | 0.88 | 0.85 |
| 3 | 4.24 | 0.94 | 1.25 |
| 4 | 4.42 | 1.16 | 1.74 |
| 5 | 4.71 | 1.16 | 1.88 |
| 6 | 5 | 1.47 | 2.31 |
| 7 | 5.1 | 1.47 | 2.41 |
| 8 | 5.28 | 1.57 | 2.61 |
| 9 | 6.29 | 1.61 | 2.83 |
| 10 | 6.67 | 1.62 | 3.35 |
| 11 | 6.83 | 1.68 | 3.36 |
| 12 | 7.64 | 1.81 | 4.9 |
| 13 | 8.1 | 2.34 | 5.07 |
| 14 | 11 | 2.9 | 5.46 |
| 15 | 13.8 | 3 | 5.64 |
| **Mean** | **6.43** | **1.631** | **2.960** |

| **Comparison** | **P-Value** |
| --- | --- |
| WT vs *ced-1* | 8.90E-06 |
| WT vs *dyn-1* | 0.00046509 |
