# Supplementary material for: Autophagosomes fuse to phagosomes and facilitate the degradation of apoptotic cells in Caenorhabditis elegans
Source: eLife. 2022 Jan 4;11:e72466. doi: 10.7554/eLife.72466 (PMC8769646; doi:10.7554/eLife.72466)
Supplement: Figure 10—source data 1. [file elife-72466-fig10-data1.docx]

**Numerical data for figure 10D – mCherry::LGG-1 signal intensity over time.**

|  | **Genotype** | | | |
| --- | --- | --- | --- | --- |
| **Time (min)** | **Wild-Type** | ***ced-6 (n2095)*** | ***ced-5 (n1812)*** | ***ced-10 (n1993)*** |
| 0 | 1 | 1 | 1 | 1 |
| 2 | 0.85 | 0.67 | 1.19 | 0.65 |
| 4 | 0.85 | 0.27 | 1 | 0.85 |
| 6 | 0.74 | 0.53 | 0.9 | 0.54 |
| 8 | 0.59 | 0.93 | 1.1 | 0.92 |
| 10 | 0.63 | 0.67 | 1.05 | 0.88 |
| 12 | 0.85 | 0.13 | 0.95 | 0.77 |
| 14 | 0.63 | 0.6 | 2.62 | 0.92 |
| 16 | 2.22 | 0.93 | 2.62 | 1.46 |
| 18 | 1.96 | 0.87 | 2.29 | 1.96 |
| 20 | 1.96 | 0.67 | 2.29 | 1.96 |
| 22 | 2 | 0.4 | 2.95 | 1.62 |
| 24 | 2.33 | 0.47 | 3.19 | 1.85 |
| 26 | 2.41 | 0.67 | 3.62 | 2.19 |
| 28 | 2.74 | 0.53 | 4.05 | 2.12 |
| 30 | 2.78 | 1.27 | 5.05 | 2.04 |
| 32 | 2.85 | 0.93 | 5.81 | 2.54 |
| 34 | 3.3 | 0.67 | 6.43 | 2.81 |
| 36 | 3.67 | 0.93 | 6.19 | 2.96 |
| 38 | 4.59 | 0.73 | 6.95 | 2.85 |
| 40 | 4.96 | 0.27 | 6.76 | 3.27 |
| 42 | 4.74 | 0.33 | 6.57 | 3.19 |
| 44 | 4.7 | 1.13 | 7.24 | 3.46 |
| 46 | 4.89 | 0.33 | 7.33 | 3.54 |
| 48 | 5.56 | 1.13 | 7.29 | 4 |
| 50 | 6.26 | 0.73 | 7.38 | 4.42 |

**Numerical data for figure 10I – mCherry::LGG-2 signal intensity over time.**

|  | **Genotype** | | | |
| --- | --- | --- | --- | --- |
| **Time (min)** | **Wild-Type** | ***ced-6 (n2095)*** | ***ced-5 (n1812)*** | ***ced-10 (n1993)*** |
| 0 | 1 | 1 | 1 | 1 |
| 2 | 1.67 | 0.69 | 0.83 | 0.25 |
| 4 | 2.19 | 0.81 | 0.64 | 1.42 |
| 6 | 1.66 | 1.11 | 1.06 | 1 |
| 8 | 1.34 | 0.76 | 1.03 | 0.42 |
| 10 | 1.08 | 0.84 | 0.89 | 2.08 |
| 12 | 1.27 | 0.81 | 1.06 | 2.42 |
| 14 | 1.21 | 0.74 | 2.58 | 6.42 |
| 16 | 1.98 | 0.98 | 2.25 | 7.75 |
| 18 | 1.88 | 0.63 | 2.94 | 8.92 |
| 20 | 2.57 | 0.61 | 3.06 | 10 |
| 22 | 2.54 | 0.63 | 4 | 10.25 |
| 24 | 3.19 | 0.56 | 4.06 | 10.17 |
| 26 | 3.39 | 0.71 | 4.28 | 11.42 |
| 28 | 3.7 | 0.45 | 4.81 | 10.67 |
| 30 | 4.34 | 0.74 | 4.89 | 10.92 |
| 32 | 4.43 | 0.69 | 4.89 | 11.58 |
| 34 | 5.22 | 0.87 | 4.81 | 11.5 |
| 36 | 5.13 | 0.89 | 6.5 | 14.33 |
| 38 | 5.38 | 0.48 | 5.64 | 12.08 |
| 40 | 5.46 | 1.02 | 6.61 | 15.33 |
| 42 | 6.41 | 0.61 | 6.44 | 13.92 |
| 44 | 6.53 | 0.61 | 8.78 | 11.83 |
| 46 | 7.76 | 0.55 | 8.14 | 16.17 |
| 48 | 8.13 | 0.47 | 10.33 | 16.75 |
| 50 | 8.17 | 0.48 | 12.47 | 18.42 |
