# Supplementary material for: Autophagosomes fuse to phagosomes and facilitate the degradation of apoptotic cells in Caenorhabditis elegans
Source: eLife. 2022 Jan 4;11:e72466. doi: 10.7554/eLife.72466 (PMC8769646; doi:10.7554/eLife.72466)
Supplement: Figure 10—source data 2. [file elife-72466-fig10-data2.docx]

**Numerical data and statistical analysis for figure 10E – Relative mCherry::LGG-1 signal intensity 50min-post engulfment.**

|  | **Genotype** | | | |
| --- | --- | --- | --- | --- |
| **Sample** | **Wild-Type** | ***ced-6 (n2095)*** | ***ced-5 (n1812)*** | ***ced-10 (n1993)*** |
| 1 | 3.5 | 1.92 | 4.16 | 3.68 |
| 2 | 3.6 | 1.40 | 4.74 | 4.92 |
| 3 | 3.8 | 1.33 | 4.88 | 5.36 |
| 4 | 4.6 | 3.00 | 5.3 | 5.66 |
| 5 | 4.7 | 5.78 | 5.49 | 5.74 |
| 6 | 4.9 | 0.72 | 5.66 | 6.72 |
| 7 | 4.9 | 0.14 | 5.92 | 6.76 |
| 8 | 4.9 | 0.62 | 5.98 | 7.3 |
| 9 | 5.8 | 2.38 | 6.49 | 7.71 |
| 10 | 7.3 | 1.40 | 6.72 | 7.94 |
| 11 | 7.4 | 2.75 | 6.75 | 9.24 |
| 12 | 8.5 | 0.89 | 6.82 | 9.97 |
| 13 | 8.9 | 0.86 | 6.85 | 10.44 |
| 14 | 9.9 | 2.33 | 7.99 | 11.91 |
| 15 | 13.9 | 0.50 | 8.21 | 17.8 |
| **Mean** | **6.44** | **1.734** | **6.130** | **8.076** |

| **Comparison** | **P-Value** |
| --- | --- |
| WT vs *ced-6* | 1.5414E-05 |
| WT vs *ced-5* | 0.70478388 |
| WT vs *ced-10* | 0.17453132 |

**Numerical data and statistical analysis for figure 10J – Relative mCherry::LGG-2 signal intensity 50min-post engulfment.**

|  | Genotype | | | |
| --- | --- | --- | --- | --- |
| **Sample** | **Wild-Type** | ***ced-6 (n2095)*** | ***ced-5 (n1812)*** | ***ced-10 (n1993)*** |
| 1 | 3.1 | 0.58 | 3.76 | 3.65 |
| 2 | 3.44 | 0.7 | 4.53 | 3.65 |
| 3 | 3.8 | 0.87 | 4.59 | 4.57 |
| 4 | 3.8 | 0.9 | 4.75 | 4.88 |
| 5 | 4 | 0.91 | 5.28 | 5.08 |
| 6 | 4.34 | 0.92 | 5.66 | 5.64 |
| 7 | 4.4 | 0.94 | 6.68 | 5.87 |
| 8 | 5.1 | 1 | 6.69 | 6.37 |
| 9 | 5.4 | 1.06 | 7.51 | 6.5 |
| 10 | 5.6 | 1.4 | 7.51 | 6.76 |
| 11 | 7.4 | 1.56 | 11.31 | 7.19 |
| 12 | 9 | 1.71 | 12.21 | 9 |
| 13 | 11.8 | 2.03 | 12.63 | 9.38 |
| 14 | 12.4 | 2.39 | 13.85 | 13.7 |
| 15 | 14 | 2.6 | 14.3 | 13.72 |
| **Mean** | **6.505** | **1.304** | **8.084** | **7.064** |

| **Comparison** | **P-Value** |
| --- | --- |
| WT vs *ced-6* | 5.8918E-05 |
| WT vs *ced-5* | 0.24675665 |
| WT vs *ced-10* | 0.6548804 |
