# Supplementary material for: Autophagosomes fuse to phagosomes and facilitate the degradation of apoptotic cells in Caenorhabditis elegans
Source: eLife. 2022 Jan 4;11:e72466. doi: 10.7554/eLife.72466 (PMC8769646; doi:10.7554/eLife.72466)
Supplement: Figure 11—source data 1. [file elife-72466-fig11-data1.docx]

**Numerical data for Figure 11E - NUC-1::mCherry signal intensity over time.**

|  | **Genotype** | | | |
| --- | --- | --- | --- | --- |
| **Time (min)** | **Wild-Type** | ***lgg-1 (tm3489)*** | ***lgg-2 (tm5755)*** | ***atg-7 (bp411)*** |
| **0** | 1 | 1 | 1 | 1 |
| **4** | 1.172 | 0.9 | 1.01 | 0.89 |
| **8** | 1.227 | 0.7 | 1.04 | 0.91 |
| **12** | 1.205 | 0.64 | 0.93 | 1.32 |
| **16** | 1.282 | 0.65 | 1.65 | 1.89 |
| **20** | 2.125 | 1.09 | 1.88 | 1.91 |
| **24** | 2.311 | 1.35 | 1.77 | 1.56 |
| **28** | 2.817 | 1.64 | 1.77 | 1.63 |
| **32** | 3.121 | 1.67 | 2.03 | 0.61 |
| **36** | 3.883 | 1.85 | 2.84 | 2.36 |
| **40** | 3.641 | 1.91 | 2.84 | 2.51 |
| **44** | 4 | 2.42 | 3.25 | 2.47 |
| **48** | 4.938 | 2.99 | 3.5 | 2.87 |
| **52** | 5.3 | 2.71 | 4.22 | 4.17 |
| **60** | 6.22 | 4.96 | 6.57 | 5.51 |
