# Supplementary material for: Autophagosomes fuse to phagosomes and facilitate the degradation of apoptotic cells in Caenorhabditis elegans
Source: eLife. 2022 Jan 4;11:e72466. doi: 10.7554/eLife.72466 (PMC8769646; doi:10.7554/eLife.72466)
Supplement: Figure 11—source data 2. [file elife-72466-fig11-data2.docx]

**Numerical data and statistical analysis for Figure 11F - Relative NUC-1::mCherry at 60 min-post engulfment.**

|  | **Genotype** | | | |
| --- | --- | --- | --- | --- |
| **Sample** | **Wild-Type** | ***lgg-1 (tm3489)*** | ***lgg-2 (tm5755)*** | ***atg-7 (bp411)*** |
| 1 | 2.864 | 2.824 | 2.110 | 3.228 |
| 2 | 2.875 | 2.982 | 2.537 | 3.406 |
| 3 | 3.221 | 3.280 | 3.042 | 3.684 |
| 4 | 3.287 | 3.873 | 3.100 | 3.817 |
| 5 | 3.335 | 3.913 | 3.101 | 3.991 |
| 6 | 3.455 | 3.931 | 3.464 | 4.028 |
| 7 | 3.478 | 4.281 | 4.159 | 5.121 |
| 8 | 3.794 | 4.474 | 4.466 | 5.157 |
| 9 | 3.975 | 4.691 | 4.552 | 5.577 |
| 10 | 4.685 | 5.617 | 4.655 | 5.676 |
| 11 | 5.587 | 5.969 | 4.761 | 5.973 |
| 12 | 5.868 | 6.284 | 4.767 | 6.087 |
| 13 | 7.121 | 6.317 | 4.803 | 6.170 |
| 14 | 7.711 | 6.750 | 6.084 | 6.315 |
| 15 | 7.738 | 6.852 | 6.125 | 6.582 |
| **Mean** | **4.600** | **4.803** | **4.115** | **4.987** |

| **Comparison** | **P-Value** |
| --- | --- |
| WT vs *lgg-1* | 0.7282830 |
| WT vs *lgg.2* | 0.3859367 |
| WT vs *atg-7* | 0.4842739 |

**Numerical data and statistical analysis for Figure 11G – Time when NUC-1 fusion starts.**

|  | Genotype | | | |
| --- | --- | --- | --- | --- |
| **Sample** | **Wild-Type** | ***lgg-1 (tm3489)*** | ***lgg-2 (tm5755)*** | ***atg-7 (bp411)*** |
| 1 | 10 | 10 | 8 | 8 |
| 2 | 12 | 12 | 10 | 10 |
| 3 | 14 | 12 | 10 | 10 |
| 4 | 14 | 12 | 10 | 10 |
| 5 | 14 | 14 | 12 | 12 |
| 6 | 14 | 14 | 12 | 12 |
| 7 | 14 | 14 | 12 | 12 |
| 8 | 14 | 14 | 12 | 12 |
| 9 | 16 | 14 | 14 | 12 |
| 10 | 16 | 16 | 14 | 14 |
| 11 | 16 | 18 | 16 | 14 |
| 12 | 18 | 18 | 16 | 14 |
| 13 | 18 | 22 | 16 | 16 |
| 14 | 18 | 24 | 18 | 16 |
| 15 | 22 | 24 | 18 | 26 |
| **Mean** | **15.333** | **15.867** | **13.200** | **13.200** |

| **Comparison** | **P-Value** |
| --- | --- |
| WT vs *lgg-1* | 0.7001003 |
| WT vs *lgg.2* | 0.0614423 |
| WT vs *atg-7* | 0.1175689 |
