# Supplementary material for: Autophagosomes fuse to phagosomes and facilitate the degradation of apoptotic cells in Caenorhabditis elegans
Source: eLife. 2022 Jan 4;11:e72466. doi: 10.7554/eLife.72466 (PMC8769646; doi:10.7554/eLife.72466)
Supplement: Figure 12—source data 1. [file elife-72466-fig12-data1.docx]

**Numerical data for Figure 12C – Percentage distribution percentage of autolysosomes (GFP^+^ mCherry^+^ puncta) among LGG-1^+^ or -2^+^ puncta on the surface of C1, C2, and C3 phagosomes.**

|  | **% of Puncta population** | |
| --- | --- | --- |
| **Sample** | **LGG-1^+^ NUC-1+** | **LGG-2^+^**  **NUC-1^+^** |
| 1 | 41.176 | 38.235 |
| 2 | 46.667 | 40.000 |
| 3 | 41.176 | 29.412 |
| 4 | 31.034 | 44.828 |
| 5 | 33.333 | 50.000 |
| 6 | 33.333 | 38.095 |
| 7 | 36.364 | 31.818 |
| **Mean** | **40.69** | **36.50** |
| **SD** | **10.83** | **5.05** |
