# Supplementary material for: Autophagosomes fuse to phagosomes and facilitate the degradation of apoptotic cells in Caenorhabditis elegans
Source: eLife. 2022 Jan 4;11:e72466. doi: 10.7554/eLife.72466 (PMC8769646; doi:10.7554/eLife.72466)
Supplement: Figure 13—source data 1. [file elife-72466-fig13-data1.docx]

**Numerical data for Figure 13D – The acidification index curves of three phagosomes over time.**

|  | **Genotype** | | |
| --- | --- | --- | --- |
| **Time (min)** | **Wild-Type** | ***cup-5 (n3265)*** | ***atg-7 (bp411)*** |
| 0 | 1 | 1.000 | 1 |
| 3 | 0.89 | 0.987 | 0.896 |
| 6 | 0.891 | 0.961 | 0.875 |
| 9 | 0.673 | 0.937 | 0.875 |
| 12 | 0.736 | 0.967 | 0.863 |
| 15 | 0.661 | 0.956 | 0.787 |
| 18 | 0.652 | 0.921 | 0.954 |
| 21 | 0.658 | 0.913 | 0.853 |
| 24 | 0.659 | 0.938 | 0.769 |
| 27 | 0.643 | 0.914 | 0.714 |
| 30 | 0.589 | 0.914 | 0.677 |
| 33 | 0.551 | 0.911 | 0.704 |
| 36 | 0.488 | 0.891 | 0.701 |
| 39 | 0.468 | 0.917 | 0.686 |
| 42 | 0.443 | 0.909 | 0.696 |
| 45 | 0.383 | 0.884 | 0.643 |
| 48 | 0.402 | 0.907 | 0.621 |
| 51 | 0.38 | 0.934 | 0.579 |
| 54 |  | 0.938 | 0.487 |
| 57 |  | 0.918 | 0.479 |
| 60 |  | 0.888 | 0.425 |
