# Supplementary material for: Autophagosomes fuse to phagosomes and facilitate the degradation of apoptotic cells in Caenorhabditis elegans
Source: eLife. 2022 Jan 4;11:e72466. doi: 10.7554/eLife.72466 (PMC8769646; doi:10.7554/eLife.72466)
Supplement: Figure 13—source data 2. [file elife-72466-fig13-data2.docx]

**Source data for Figure 13E - distribution of the acidification index measured at 51-min-post the formation of nascent phagosomes.**

|  | **Genotype** | | |
| --- | --- | --- | --- |
| **Sample** | **Wild-Type** | ***cup-5 (n3265)*** | ***atg-7 (bp411)*** |
| 1 | 0.314 | 0.698 | 0.421 |
| 2 | 0.387 | 0.753 | 0.519 |
| 3 | 0.410 | 0.787 | 0.543 |
| 4 | 0.422 | 0.825 | 0.589 |
| 5 | 0.541 | 0.827 | 0.601 |
| 6 | 0.587 | 0.841 | 0.605 |
| 7 |  | 0.892 | 0.627 |
| 8 |  | 0.940 | 0.710 |
| 9 |  | 1.012 | 0.771 |
| 10 |  | 1.037 | 0.771 |
| 11 |  | 1.051 | 0.804 |
| 12 |  | 1.064 | 0.891 |
| 13 |  | 1.131 | 0.975 |
| **Mean** | **0.443** | **0.912** | **0.679** |
| **Min** | **0.314** | **0.698** | **0.421** |
| **Max** | **0.587** | **1.131** | **0.975** |
| **SD** | **0.102** | **0.137** | **0.158** |
| **P-Value Compared to WT** |  | **1.3657E-06** | **0.0014435** |
