# Supplementary material for: Autophagosomes fuse to phagosomes and facilitate the degradation of apoptotic cells in Caenorhabditis elegans
Source: eLife. 2022 Jan 4;11:e72466. doi: 10.7554/eLife.72466 (PMC8769646; doi:10.7554/eLife.72466)
Supplement: Figure 14—source data 1. [file elife-72466-fig14-data1.docx]

**Numerical data for Figure 14D – The ratio of the nuclear diameter curves of three phagosomes over time.**

|  | **Genotype** | | |
| --- | --- | --- | --- |
| **Time (min)** | **Wild-Type** | ***cup-5 (n3265)*** | ***atg-7 (bp411)*** |
| 0 | 1 | 1 | 1 |
| 3 | 1 | 1 | 0.949 |
| 6 | 0.956 | 0.949 | 0.967 |
| 9 | 0.892 | 0.961 | 0.956 |
| 12 | 0.819 | 0.936 | 0.874 |
| 15 | 0.783 | 0.982 | 0.895 |
| 18 | 0.679 | 1 | 1.054 |
| 21 | 0.811 | 0.924 | 1.054 |
| 24 | 0.88 | 0.961 | 0.964 |
| 27 | 0.853 | 1 | 0.979 |
| 30 | 0.877 | 0.971 | 0.949 |
| 33 | 0.957 | 0.92 | 0.956 |
| 36 | 0.921 | 1 | 0.909 |
| 39 | 0.892 | 0.924 | 0.823 |
| 42 | 0.818 | 1 | 0.733 |
| 45 | 0.848 | 1.009 | 0.893 |
| 48 | 0.799 | 0.949 | 0.9 |
| 51 | 0.779 | 0.898 | 0.845 |
| 54 | 0.719 | 0.949 | 0.774 |
| 57 | 0.663 | 0.997 | 0.692 |
| 60 | 0.495 | 0.936 | 0.646 |
