# Supplementary material for: Autophagosomes fuse to phagosomes and facilitate the degradation of apoptotic cells in Caenorhabditis elegans
Source: eLife. 2022 Jan 4;11:e72466. doi: 10.7554/eLife.72466 (PMC8769646; doi:10.7554/eLife.72466)
Supplement: Figure 14—source data 2. [file elife-72466-fig14-data2.docx]

**Source data for Figure 14E - distribution of the ratio of nuclear diameters measured at 60min-post phagosome formation.**

|  | **Genotype** | | |
| --- | --- | --- | --- |
| **Sample** | **Wild-Type** | ***atg-7 (bp411)*** | ***cup-5 (n3265)*** |
| 1 | 0.291 | 0.444 | 0.766 |
| 2 | 0.332 | 0.558 | 0.779 |
| 3 | 0.369 | 0.580 | 0.803 |
| 4 | 0.460 | 0.587 | 0.831 |
| 5 | 0.472 | 0.599 | 0.877 |
| 6 | 0.479 | 0.609 | 0.933 |
| 7 | 0.495 | 0.625 | 0.975 |
| 8 | 0.624 | 0.631 | 0.990 |
| 9 | 0.741 | 0.672 | 1.035 |
| 10 |  | 0.832 |  |
| 11 |  | 0.838 |  |
| 12 |  | 0.971 |  |
| **Mean** | **0.473** | **0.662** | **0.888** |
| **Min** | **0.291** | **0.444** | **0.766** |
| **Max** | **0.741** | **0.971** | **1.035** |
| **SD** | **0.141** | **0.146** | **0.099** |
| **P-Value**  **Compared to WT** |  | **0.00018737** | **5.7349E-08** |
